# Grape Pomace Polyphenolic Extract Promotes Osteogenic Differentiation in Human Mesenchymal Stem Cells Through Activation of RUNX2 and NRF2 Transcription Factors: A Potential Natural Strategy for Osteoporosis Prevention

**DOI:** 10.3390/biology15090719

**Published:** 2026-05-01

**Authors:** Nadia Calabriso, Marika Massaro, Stefano Quarta, Luisa Siculella, Giuseppe Santarpino, Tiziano Verri, Carmela Gerardi, Giovanna Giovinazzo, Maria Annunziata Carluccio

**Affiliations:** 1 Institute of Clinical Physiology (IFC), National Research Council (CNR), 73100 Lecce, Italy; marika.massaro@cnr.it (M.M.); stefanoquarta@cnr.it (S.Q.); mariaannunziata.carluccio@cnr.it (M.A.C.); 2 Department of Experimental Medicine (DiMeS), University of Salento, 73100 Lecce, Italy; luisa.siculella@unisalento.it; 3 Department of Clinical and Experimental Medicine, Magna Graecia University of Catanzaro, 88100 Catanzaro, Italy; santarpino@unicz.it; 4 Department of Biological and Environmental Sciences and Technologies (DISTEBA), University of Salento, 73100 Lecce, Italy; tiziano.verri@unisalento.it; 5 Institute of Sciences of Food Production (ISPA), National Research Council (CNR), 73100 Lecce, Italy; carmela.gerardi@cnr.it (C.G.); giovanna.giovinazzo@cnr.it (G.G.)

**Keywords:** grape pomace, polyphenols, osteogenic differentiation, adipogenic differentiation, human mesenchymal stem cells, adipose-derived mesenchymal stem cells, transcription factors, RUNX2, NRF2

## Abstract

Osteoporosis is an age-related metabolic bone disorder characterized by an imbalance between bone resorption and formation. Natural polyphenols have gained attention as potential complementary strategies for its prevention. In this study, we investigated the effects of a sustainable, polyphenol-rich extract from red grape pomace (GPE) on human mesenchymal stem cell (MSC) fate and its underlying mechanisms of action. We found that GPE significantly promoted osteogenic differentiation while suppressing adipogenic differentiation in canonical bone marrow-derived MSCs (BMSCs). This biological effect was preserved in adipose tissue-derived MSCs (AdMSCs) obtained from elderly patients (>65 years) at high cardiovascular risk. Mechanistically, GPE downregulated adipogenic markers (PPARγ, CD36 and FABP4) and enhanced osteogenic markers (RUNX2, ALP, OSX, BMP-2, OPN, COL1A1 and OCN). Moreover, GPE activated NRF2-dependent redox signaling, as evidenced by increased NRF2 nuclear translocation and transcriptional activity. Accordingly, GPE treatment significantly upregulated, or consistently increased, the expression of multiple NRF2 target genes, including HO-1, GPX, CAT, GCLC, and NQO1. Importantly, pharmacological inhibition of NRF2 attenuated GPE-induced ALP activity, confirming NRF2 as a key mediator of its osteogenic effects. Overall, grape pomace-derived polyphenols act as upstream modulators of redox-sensitive and osteogenic transcription factors, rebalancing MSC differentiation toward osteogenesis and mitigating age-related bone fragility.

## Simple Summary

Osteoporosis is an age-related condition characterized by reduced bone formation and increased bone loss. Natural polyphenols are being studied as potential supportive strategies for bone health. In this study, we evaluated the effects of a polyphenol-rich extract from red grape pomace (GPE) on human mesenchymal stem cells. GPE promoted their differentiation into bone-forming cells while reducing their tendency to become fat cells, without affecting cell viability. These effects were associated with increased expression of key osteogenic markers and activation of the antioxidant transcription factor NRF2. Blocking NRF2 reduced the osteogenic effect of GPE, highlighting its central role. Overall, grape pomace-derived polyphenols emerge as a potent modulator of the fate of mesenchymal stem cells, offering a novel strategy to restore osteogenic balance and mitigate age-related bone fragility.

## 1. Introduction

Osteoporosis is a systemic skeletal disorder characterized by low bone mass, deterioration of bone microarchitecture, and increased bone fragility, leading to a heightened risk of fractures [1]. With the progressive aging of the global population, osteoporosis has emerged as a major public health concern and one of the most prevalent non-communicable diseases, significantly impairing quality of life, particularly in postmenopausal women. Consequently, identifying effective, sustainable, and globally applicable preventive strategies is of critical importance [2]. Osteoporosis arises from an imbalance between bone resorption and formation, driven by excessive osteoclast activity and impaired osteoblast function [1]. Osteoblasts, which originate from mesenchymal stem cells (MSCs), are responsible for bone matrix synthesis and mineral deposition, whereas osteoclasts, derived from the hematopoietic monocyte/macrophage lineage, are specialized in the resorption of mineralized bone matrix.

Bone repair and remodeling are finely regulated processes that depend on a balanced interplay between osteoblastogenesis and osteoclastogenesis. The osteogenic differentiation of bone marrow-derived mesenchymal stem cells (BMSCs) is regulated by several bone morphogenetic proteins (BMPs) and Wnt signaling pathways [3]. Active osteoblasts synthesize and secrete type I collagen (COLI), the principal protein of the bone matrix, as well as non-collagenous proteins such as osteocalcin (OCN), osteopontin (OPN), and osteonectin. They are also characterized by high alkaline phosphatase (ALP) activity, a well-established biomarker of osteoblastic function that plays a crucial role in bone matrix mineralization. In addition, osteoblasts regulate osteoclastogenesis through the Wnt signaling pathway and by secreting osteoprotegerin (OPG) and receptor activator of nuclear factor κB ligand (RANKL). RANKL induces reactive oxygen species (ROS), which, acting as intracellular mediators of osteoclastogenesis, promote osteoclast differentiation. While physiological ROS levels contribute to bone remodeling, excessive ROS disrupt this balance by enhancing osteoclastogenesis and inhibiting osteoblast function, ultimately leading to bone loss and osteoporosis. Oxidative stress also impairs the activity of nuclear factor erythroid 2-related factor 2 (NRF2), a key regulator of antioxidant defense and bone homeostasis. Reduced NRF2 expression, as observed under oxidative stress and in osteoporotic models, favors osteoclast formation and further contributes to bone deterioration. NRF2 activation also plays a significant role in osteogenic differentiation [3]. Besides preserving the stemness of mesenchymal stem cells (MSCs), it promotes mineralization by modulating the activity of Runt related transcription factor 2 (RUNX2), a key regulator of osteogenic differentiation [3]. Of note, NRF2 is also a pivotal transcription factor that regulates cytoprotective and antioxidant genes by binding to antioxidant response elements (AREs) [3]. Under basal conditions, NRF2 is negatively regulated by Kelch-like ECH-associated protein 1 (KEAP1), which promotes its ubiquitination and proteasomal degradation. In response to oxidative stress, modifications of KEAP1 reduce its affinity for NRF2, allowing NRF2 to dissociate from KEAP1, translocate to the nucleus, and activate target genes such as NAD(P)H quinone dehydrogenase 1 (NQO1), heme oxygenase-1 (HO-1), and glutathione S-transferase (GST). Moreover, oxidative stress–induced activation of FOXO transcription factors enhances the expression and activity of peroxisome proliferator-activated receptor gamma (PPARγ), which promotes adipogenesis while inhibiting osteogenesis [4]. Indeed, aging-related bone loss is accompanied by increased bone marrow adiposity, reflecting a shift in MSC fate toward adipogenesis at the expense of osteoblastogenesis [5].

Recent studies suggested that natural bioactive compounds, counteracting oxidative stress, may promote bone health, and could be beneficial in preventing bone loss, as well as in reducing the onset and progression of osteoporosis and the associated fracture risk [6].

Polyphenols are plant-derived bioactive compounds with well-documented health benefits [7,8,9]. Increasing evidence indicates that higher polyphenol intake may slow osteoporosis progression by enhancing bone anabolic activity [7]. Large population studies associate higher fruit and polyphenol intake—particularly anthocyanins abundant in grapes—with increased bone mineral density and reduced fracture risk, highlighting that diets rich in polyphenols protected bone from age-related and postmenopausal loss [8,9].

Grape pomace, a winemaking byproduct, is an exceptionally rich source of polyphenols and other bioactive compounds. Owing to its strong biological activity, grape pomace represents a promising and sustainable natural resource for the prevention and management of age-related disorders such as osteoporosis [10].

Our research group has previously demonstrated that grape pomace extracts and their isolated polyphenols exert vascular-protective effects by attenuating intracellular oxidative stress, inflammation, and pathological angiogenesis in vascular cells [11,12,13]. Given the well-established functional and molecular crosstalk between the vascular and skeletal systems [13], these findings suggest that grape pomace polyphenols may modulate shared pathogenic pathways involved in both vascular dysfunction and bone loss, providing a strong rationale for investigating their effects on bone metabolism and osteogenic differentiation.

Negroamaro, a *Vitis vinifera* cultivar that is native to the Salento area of Southern Italy and widely used in local winemaking, is characterized by a peculiar polyphenolic content [14].

Within the PRIN-2022 PNRR project WINPROAGE (Wine by-products metabolomic profiling in natural chemoprevention of vascular ageing), developed in the framework of a circular economy and a One Health approach, eco-sustainable and low-cost processes were developed to valorize Negroamaro grape pomace by producing a polyphenol-rich beverage with potential nutraceutical applications.

In this context, the present study investigates the effects of a polyphenol-rich grape pomace extract (GPE), obtained by aqueous infusion, on osteogenic and adipogenic differentiation of BMSCs. Given the shared involvement of inflammation and oxidative stress in both cardiovascular disease and osteoporosis, we also evaluated the effects of GPE on MSCs derived from adipose tissue (AdMSCs) of elderly subjects (>65 years) at high cardiovascular risk.

Finally, we investigated the underlying mechanisms of action of GPE by analyzing the expression and activation of two key transcription factors involved in osteogenesis: RUNX2, which is essential for osteoblast differentiation and bone formation, and NRF2, a central regulator of oxidative stress-related signaling pathways involved in bone metabolism. Overall, this work aims to support the GPE potential as a nutraceutical strategy for the prevention of age-related disorders such as osteoporosis.

## 2. Materials and Methods

### 2.1. Chemicals

Type 2 collagenase was obtained from Worthington Biochemicals (Lakewood, NJ, USA). ML385 was obtained from Cayman Chemical Company (Ann Arbor, MI, USA). Unless otherwise indicated, all other reagents were purchased from Merck Life Science (Milan, Italy).

### 2.2. Raw Material and Sample Preparation

Three batches of wine pomace, *Vitis vinifera* cv Negroamaro (achieved after fermentation for red wine making), were obtained from a commercial winemaking facility located in Salento (southern area of the Apulia Region, Italy). The pomace was dried in an oven at 50 °C until it reached a constant weight. We obtained grape pomace sample by infusing Negroamaro grape pomace (10 g) in 100 mL of distilled water acidified with 1% of citric acid 1 M at 90 °C for 5 min in the dark. Cell debris were removed by centrifugation (4000× *g*) for 5 min. After which, the sample was dried with a spray dryer (MiniSprayDryer S-300 BUCHI, Flawil, Switzerland). Subsequently a resuspension of dried grape pomace infusion was carried out in ethanol 70% and grape pomace extract (GPE) was used for further chemical characterization and biological analysis.

### 2.3. Antioxidant Activity of GPE

Trolox Equivalent Antioxidant Capacity (TEAC) Assay was performed by the method described by Re et al. [15], modified as reported by Gerardi et al. [10]. Briefly, the ABTS radical cation was diluted in PBS (pH 7.4) to an absorbance of 0.40 at 734 nm. After the addition of 200 µL of diluted ABTS to 10 µL of Trolox standard or extract, the absorbance reading at 734 nm was taken 6 min after initial mixing using an Infinite 200 Pro plate reader (Tecan, Männedorf, Switzerland). The percentage inhibition of absorbance at 734 nm was calculated and plotted as a function of the concentration of Trolox, and the TEAC value expressed as Trolox equivalents (µmol) using Magellan v7.2 software.

### 2.4. Folin–Ciocalteu Assay

A rapid method was used to assess the total phenols in water extracts from dried pomace in 96-well plates (Corning, Glendale, AZ, USA) using a microplate reader (Tecan, Infinite M200). Folin–Ciocalteu reagent (1:5, *v*/*v*) (50 µL) was placed in each well, and then 100 µL of sodium hydroxide solution (0.35 M) was added. The absorbance at 760 nm of the blue complex formed was monitored after 5 min. Gallic acid was used to obtain a calibration curve in the range from 2.5 to 40.0 mg/L (R ≥ 0.9997). The total phenol content of the samples was expressed as gallic acid equivalents.

### 2.5. High-Performance Liquid Chromatography (HPLC) Characterization of Several Classes of Polyphenols

Different phenolic compounds present in aqueous extracts were separated by RP-HPLC DAD (Agilent 1100 HPLC system, Santa Clara, CA, USA). The separation was performed as described by Gerardi et al. [16]. The wavelengths used for quantification of phenol classes compounds were 280 for flavanols, 306 for stilbenes 320 for hydroxycinnamic acids, 370 for flavonols and 520 nm for anthocyanins. Quantitation was carried out by an external standard method, using the most abundant compound of each polyphenol class as the calibration standard: thus flavanols were quantified as catechin, stilbenes as resveratrol-3-O-glucoside, hydroxycinnamic acids as caffeic acid, flavonols as quercetin and anthocyanins as oenin.

### 2.6. Mesenchymal Stem Cell Culture

Human bone marrow-derived mesenchymal stem cells (BMSCs) from healthy donors (ATCC-PCS-500-012) were purchased from ATCC (Milan, Italy) as well as mesenchymal stem cell basal medium (ATCC PCS500030) and mesenchymal stem cell growth kit for BMSC (ATCC PCS500041). BMSC from three different lots were used to perform all experiments. Cells were used between the third and the sixth passages.

Adipose derived mesenchymal stem cells (AdMSCs) were isolated from pericardial adipose tissue obtained from patients at high cardiovascular risk undergoing coronary artery bypass graft (CABG) surgery at the Città di Lecce Hospital (Lecce, Italy), as previously reported [17]. The study was conducted in accordance with the ethical principles of the Declaration of Helsinki and was approved by the Ethics Committee of Bari, Italy (protocol no. 133, 26 February 2024). Written informed consent was obtained from all patients prior to surgery. AdMSCs were characterized as previously described [17], expanded up to passage 3, and used for subsequent adipogenic or osteogenic differentiation experiments. A total of five donors were included, and each experiment represents the mean of at least three independent donor samples.

### 2.7. MTT Cytotoxicity Assay

Cells were seeded in 96-well plates at a density of 1 × 10^3^ cells per well and allowed to adhere. After a starvation period, cells were treated with culture medium supplemented with GPE at increasing concentrations (1, 5, 10 and 25 µg GAE/mL) or NRF2 inhibitor ML365. After 24 h of treatment, the medium was replaced with thiazolyl blue tetrazolium bromide (MTT) solution (0.05 mg/mL), and cells were incubated for 3 h at 37 °C.

The MTT solution was then removed, and formazan crystals were solubilized by adding 100 µL of DMSO to each well. Absorbance was measured at 590 nm using a microplate reader (Multiskan^®^ FC, Thermo Fisher Scientific, Vantaa, Finland). Cell viability was expressed as a percentage relative to untreated control cells, according to the following formula: Cell viability (%) = OD treatment/OD control × 100.

### 2.8. Osteogenic or Adipogenic Differentiation Protocol of MSCs

**Figure 1:**
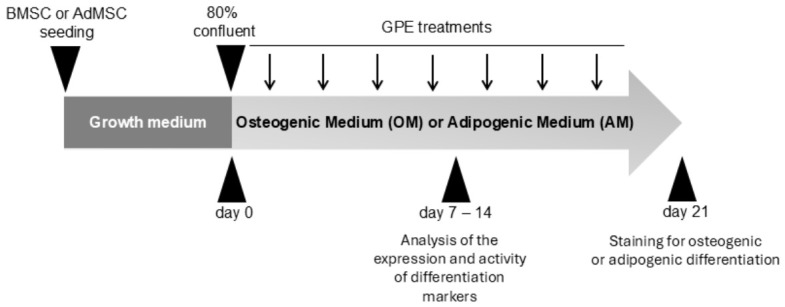
Experimental design. The arrows indicate the replacement of the differentiation medium and GPE treatment every 2–3 days.

Near-confluent cells (approximately 80% confluence) were induced to differentiate into osteoblasts or adipocytes according to the experimental scheme shown in Figure 1. Osteogenic differentiation of MSCs was induced by supplementing the culture medium with 100 nmol/L dexamethasone, 10 mmol/L β-glycerophosphate, and 100 μmol/L ascorbic acid 2-phosphate. Adipogenic differentiation of MSCs was induced by supplementing the culture medium with 500 μmol/L 3-Isobutyl-1-methylxanthine, 1 μmol/L dexamethasone, 10 μmol/L insulin and 200 μmol/L indomethacin. Both differentiation media were replaced every 2–3 days for up to 21 days. To evaluate the effect of GPE on MSC differentiation into osteoblasts or adipocytes, the experimental design involved treating MSCs with increasing concentrations of GPE (1–10 µg GAE/mL) throughout the differentiation period. As vehicle control, MSCs were incubated with an appropriate amount of solvent (<0.025% *v*/*v*). After 21 days, MSC differentiation into osteoblasts or adipocytes was assessed by staining with Alizarin Red S and Oil Red O, respectively. At intermediate differentiation times (7 and 14 days), mRNA levels of osteogenic or adipogenic differentiation markers and antioxidant enzymes were assessed by quantitative real-time PCR (qRT-PCR), as well as ALP activity was evaluated by ALP activity assay kit. In addition, the expression and activity of the transcription factor NRF2 were analyzed by RT-PCR and TransAM^®^ NRF2 kit (Active Motif, Carlsbad, CA, USA), respectively. Finally, intracellular ROS levels of MSCs were measured by carboxy-H2DCFDA staining (see following paragraphs).

### 2.9. Alizarin Red S Staining

Matrix mineralization of MSCs after 21 days of osteogenic induction was quantified using Alizarin Red S (ARS) staining, which allows for the assessment of calcium deposits. Briefly, cell cultures were washed twice with PBS, fixed with paraformaldehyde for 15 min, washed three times with distilled water, and then stained with ARS staining solution (Sigma, Merck Life Science S.r.l., Milan, Italy). Excess dye was then gently removed under running water, and the bright red calcium deposits in the matrix were analyzed under a light microscope and photographed. For quantification, the dye bound to the calcium deposits was dissolved in 10% acetic acid, and absorbance was determined at 405 nm using a microplate reader.

### 2.10. Oil Red O Staining

Oil Red O (ORO) staining (Sigma, Merck Life Science S.r.l., Milan, Italy) was used to visualize lipid droplets in MSCs after 21 days of adipocyte induction. Briefly, cell cultures were washed twice with PBS, fixed with paraformaldehyde for 15 min, and then stained with 10% (*w*/*v*) ORO in isopropanol for 20 min in the dark. Stained cells were examined under a brightfield microscope. For quantification, ORO-stained lipids were dissolved in dimethyl sulfoxide (DMSO, Sigma, Merck Life Science S.r.l., Milan, Italy), and absorbance was measured at 492 nm using a microplate reader.

### 2.11. Alkaline Phosphatase Activity

Alkaline phosphatase (ALP) activity was determined in AdMSCs induced to differentiate into osteoblasts for 7 and 14 days, using an ALP activity assay kit (Merck Life Science S.r.l., Milan, Italy). Briefly, AdMSCs were lysed with a lysis buffer consisting of 0.2% Triton X-100 in purified water, and ALP activity was determined according to the manufacturer’s instructions. The improved method uses p-nitrophenyl phosphate, which is hydrolyzed by ALP into a yellow product (maximum absorbance at 405 nm). The reaction rate, measured spectrophotometrically, is directly proportional to the enzymatic activity, which was expressed as Units/Liter.

### 2.12. RNA Isolation and Real-Time Quantitative PCR

Total RNA was extracted using the TRIzol reagent (Thermo Fisher Scientific, Waltham, MA, USA) and quantified spectrophotometrically. Total RNA (1 μg) was converted into first-strand cDNA using the High-Capacity cDNA Reverse Transcription Kit (Applied Biosystems, Monza, Italy). Quantitative RT-PCR was performed, with 16 ng cDNA, in CFX384 Touch Real-Time PCR Detection System (Bio-Rad Laboratories, Milan, Italy) using a SYBR Green PCR Master Mix (Bio-Rad Laboratories, Milan, Italy) and the synthesized primers (Thermo Fisher Scientific, Waltham, MA, USA) listed in Table 1. All reactions were assessed in triplicate. The relative changes in gene expression were calculated by comparative critical threshold method (2^−ΔΔCT^). As housekeeping gene was used, GAPDH was quantified for each sample, and the data were normalized accordingly. Results are expressed as fold increase relative to unstimulated control (=1).

### 2.13. Immunofluorescence Analysis

AdMSCs were washed three times with sterile PBS at 4 °C before being fixed in 4% (*v*/*v*) paraformaldehyde in PBS for 15 min. Cell samples were washed three times with PBS, permeabilized with PBS at pH 7.4 containing 0.1% (*v*/*v*) Triton X-100 and 1% (*w*/*v*) BSA for 30 min, and then incubated with 2.5% BSA in PBS for another 30 min to block nonspecific binding sites.

AdMSCs were incubated for 1 h at room temperature with either anti-RUNX2 or anti-osteocalcin primary antibodies. After three washes with PBS, cells were incubated for 1 h in the dark with the corresponding secondary antibodies: Alexa Fluor 488-conjugated goat anti-mouse (Bethyl Laboratories Inc., Cambridge, UK) for RUNX2, or Cy3-conjugated goat anti-rabbit (Proteintech, Manchester, UK) for osteocalcin.

Appropriate controls were performed with AdMSCs incubated without the first antibody. Immune complexes were visualized with the ZOE Fluorescent Cell Imager (Bio-Rad Laboratories, Milan, Italy) inverted fluorescence system.

### 2.14. NRF2 Activation

Nuclear proteins were extracted from total cell lysates of MSCs induced to differentiate into osteoblasts using the Active Motif Nuclear Extract Kit (Active Motif, Carlsbad, CA, USA) according to the manufacturer’s protocol. NFR-2 binding activity was analyzed using the “TransAM” kit (Active Motif) following the manufacturer’s protocol.

### 2.15. Intracellular ROS Quantification

Intracellular ROS levels were determined using a carboxy-2,7-dichlorofluorescein diacetate (CM-H2DCFDA) probe, as described previously [18]. CM-H2DCFDA is hydrolysed in the cytosol to form the DCFH carboxylate anion. Oxidation results in the formation of fluorescent DCF, which is maximally excited at 495 nm and emits at 520 nm. MSCs were then seeded in 24-well cell culture plates (5 × 10^4^ cells/well) in basal medium. After 24 h of incubation, MSCs were induced to differentiate into osteoblasts in the absence or the presence (5 μg/mL) of GPE. MSCs cultured in basal medium were considered as undifferentiated and untreated control cells (CTR). After 3 days, cells were washed and loaded with the probe CM-H2DCFDA (10 μmol/L) for 45 min at 37 °C in the dark. After gently washing the cell monolayers twice with PBS, phenol red-free medium was added, and fluorescence was immediately monitored by microplate reader or fluorescence microscopy.

### 2.16. Statistical Analysis

Data were expressed as mean ± standard deviation (SD) of at least three independent experiments. Differences between two groups were determined by unpaired Student’s *t*-test. Multiple comparisons were performed by one-way analysis of variance (ANOVA), and individual differences were then tested by Fisher’s protected least-significant difference test after the demonstration of significant inter-group differences by ANOVA. A *p* value < 0.05 was considered statistically significant.

## 3. Results

### 3.1. Phenolic Content, Antioxidant Activity, and Cytotoxicity of GPE

Preliminary experiments were conducted to optimize extraction conditions that maximize bioactive compound yield while preserving a green and eco-sustainable process. Negroamaro grape pomace aqueous extract was obtained by aqueous infusion of primary raw material at 10% (*w*/*v*) in acidified water, followed by removal of debris and spray-drying to complete dryness. The resulting powder was resuspended in 70% ethanol to obtain the GPE, which was subsequently used for chemical characterization and biological analyses. As vehicle control, MSC were incubated with appropriate amount of solvent (<0.025% *v*/*v*). These concentrations of ethanol had no effect on any of the parameters measured in this study.

GPE exhibited a total phenolic content of 372.49 ± 12.33 mg gallic acid equivalents (GAE)/L and an antioxidant activity of 1.03 ± 0.12 mmol Trolox equivalents (TE)/L, as assessed by Folin–Ciocalteu assay and TEAC assay respectively. GPE was further characterized by profiling its major polyphenol classes. As reported in Table 2, anthocyanins represented the predominant class, followed by phenolic acids, flavanols, and stilbenes, whereas flavonols were present at lower levels.

**Figure 2:**
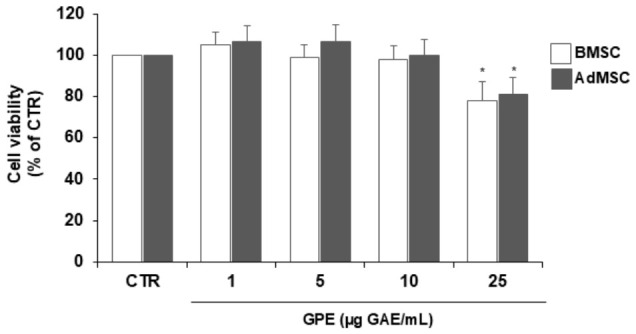
Effects of GPE on human mesenchymal stem viability. BMSC and AdMSC were exposed at increasing concentrations of GPE (1, 5, 10 and 25 µg GAE/mL) for 24 h and cell viability was evaluated by MTT assay. Data are shown as mean ± SD (*n* = 5); * *p* < 0.05 versus untreated control cells (CTR).

Before testing GPE’s effect on human MSC differentiation, its potential cytotoxicity was assessed using the MTT assay. Treatment with GPE for 24 h at concentrations up to 10 µg GAE/mL did not affect the viability of MSCs derived from either bone marrow or adipose tissue (Figure 2); therefore, these concentrations were selected for subsequent bioactivity study.

### 3.2. GPE Modulates Osteogenic and Adipogenic Differentiation of Human MSCs

Given the critical role of the balance between osteogenic and adipogenic differentiation of MSCs in the pathophysiology of osteoporosis, evaluating the effects of GPE on MSC fate is of particular relevance.

Accordingly, the first phase of the study assessed the effects of GPE on the differentiation of BMSCs obtained from healthy donors (as described in Section 2). Furthermore, considering that chronic inflammation and oxidative stress—key drivers of atherosclerotic cardiovascular disease—also contribute to osteoporosis, we investigated the effects of GPE on AdMSCs isolated from elderly (>65 years) cardiovascular patients undergoing cardiac surgery.

The aim of this study was to determine whether GPE retains its biological activity in MSCs derived from elderly high cardiovascular risk patients, despite the well-documented functional impairment of these cells.

**Figure 3:**
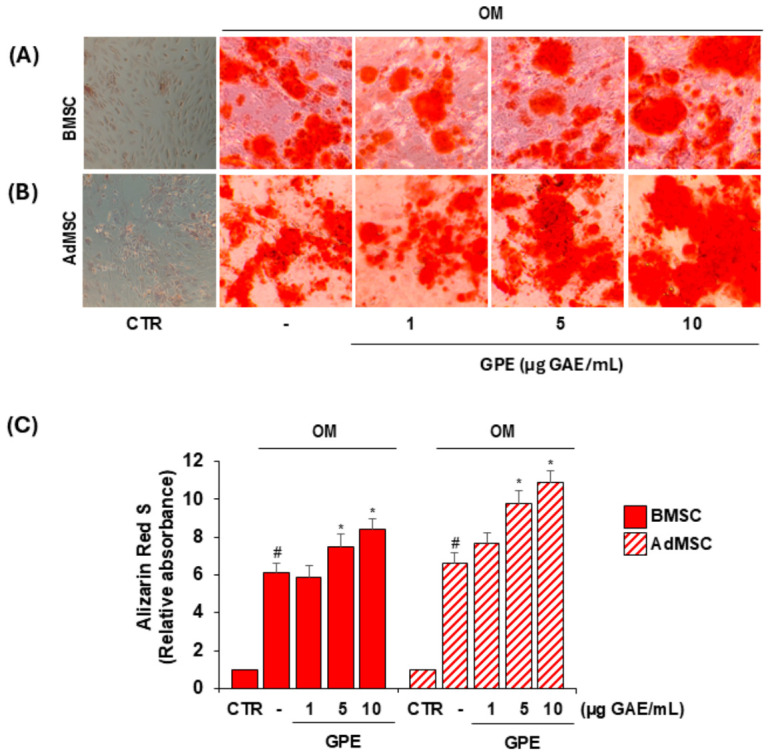
Effects of GPE on osteoblast differentiation of human MSCs. BMSCs and AdMSCs were cultured in basal medium (CTR) or osteogenic differentiation medium (OM) with or without GPE (1–10 µg GAE/mL) for up to 21 days, and matrix mineralization was analyzed by Alizarin Red S staining. Representative light microscopic images of Alizarin Red S staining in BMSCs (**A**) and AdMSCs (**B**) and quantitative analysis (**C**). Each experiment was performed in triplicate. All data are presented as mean  ±  standard deviation (SD). # *p* < 0.01 versus undifferentiated control cells (CTR); * *p* < 0.05 versus osteogenic differentiated cells (OM).

**Figure 4:**
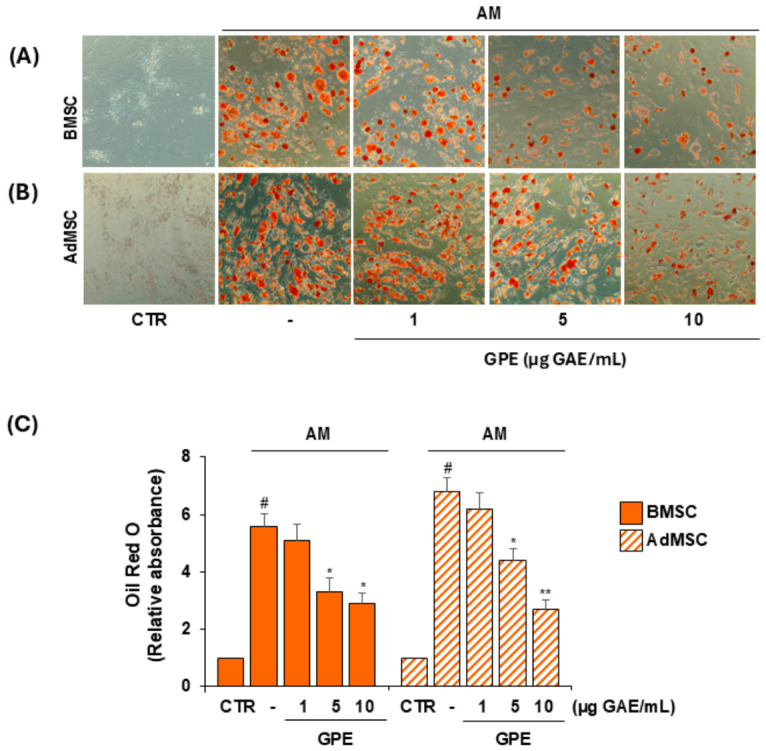
Effects of GPE on the adipogenic differentiation of human MSCs. BMSCs and AdMSCs were cultured in basal medium (CTR) or adipogenic differentiation medium (AM) with or without GPE (1–10 µg GAE/mL) for up to 21 days, and lipid droplet formation was analyzed by Oil Red O (ORO) staining. Representative light microscopic images of ORO staining in BMSCs (**A**) and AdMSCs (**B**) and quantitative analysis (**C**). Each experiment was performed in triplicate. All data are presented as mean  ±  standard deviation (SD). # *p* < 0.01 versus undifferentiated control cells (CTR); * *p* < 0.05 and ** *p* < 0.01 versus adipogenic differentiated cells (AM).

For this purpose, BMSCs and AdMSCs were induced to differentiate into osteoblasts or adipocytes using osteogenic or adipogenic medium (OM or AM, respectively), in the absence or presence of increasing concentrations of GPE (1–10 µg GAE/mL). After 21 days, osteogenic differentiation was assessed by matrix mineralization quantification using ARS staining (Figure 3), while adipogenic differentiation was evaluated by intracellular lipid droplet accumulation using ORO staining (Figure 4).

GPE modulated the differentiation potential of both BMSCs and AdMSCs, significantly enhancing osteogenesis and simultaneously suppressing adipogenesis in both cell types (Figure 3 and Figure 4). At 5 µg GAE/mL, GPE increased matrix mineralization by 25% and 48% in BMSCs and AdMSCs, respectively, compared with OM alone (Figure 3C), and reduced lipid droplet accumulation by 41% and 35%, respectively, compared with AM alone (Figure 4C).

Importantly, these effects were observed at concentrations that did not significantly affect cell viability in either BMSCs or AdMSCs, under basal conditions (Figure 2) or following osteogenic or adipogenic differentiation (Figure S1).

A concentration of 5 µg GAE/mL was selected for subsequent mechanistic studies, as it represented the lowest dose capable of significantly enhancing matrix mineralization while concurrently reducing lipid accumulation.

To further investigate the mechanism of action of GPE in osteogenic and adipogenic differentiation, most experiments were performed in AdMSCs, while key mechanisms were additionally examined in BMSCs to assess their potential dependence on cell source.

### 3.3. GPE Inhibits the Expression of Adipogenic Markers in AdMSCs

**Figure 5:**
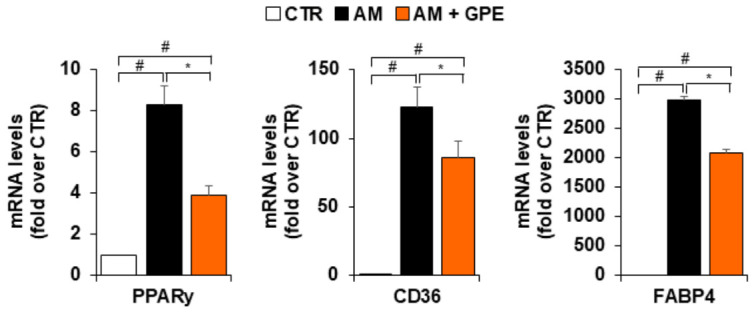
GPE’s effects on the expression of adipogenic differentiation markers. AdMSCs were cultured in basal medium (CTR) or adipogenic differentiation medium (AM) with or without GPE (5 µg GAE/mL) for 7 days. The mRNA levels of peroxisome proliferator–activated receptor gamma (PPARγ), CD36 and fatty acid–binding protein 4 (FABP4) were analyzed using quantitative real-time polymerase chain reaction (qRT-PCR). Each experiment was performed in triplicate. All data are presented as mean  ±  standard deviation (SD). # *p* < 0.01 versus undifferentiated control cells (CTR); * *p* < 0.05 versus adipogenic differentiated cells (AM).

Since GPE significantly reduced lipid droplet accumulation in MSCs undergoing adipogenic differentiation, we next examined its effects on both early and late adipogenic markers. Adipogenic medium markedly increased the mRNA levels of the key adipogenic transcription factor PPARγ; this induction was significantly reduced by approximately 63% in the presence of GPE (Figure 5). Consistently, GPE significantly downregulated the expression of CD36 and fatty acid-binding protein 4 (FABP4), two established PPARγ target genes involved in lipid uptake and intracellular fatty acid trafficking (Figure 5).

### 3.4. GPE Enhances the Expression and Activity of Osteogenic Markers in AdMSCs

**Figure 6:**
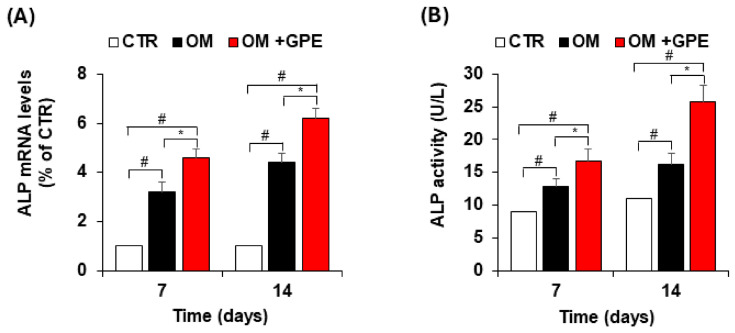
Effects of GPE on alkaline phosphatase (ALP) expression and activity in AdMSCs. AdMSCs were cultured in basal medium (CTR) or osteogenic differentiation medium (OM) with or without GPE (5 µg GAE/mL), and ALP expression (**A**) and activity (**B**) were assessed after 7 and 14 days. Each experiment was performed in triplicate. All data are presented as mean  ±  standard deviation (SD). # *p* < 0.01 versus undifferentiated control cells (CTR); * *p* < 0.05 versus osteogenic differentiated cells (OM).

Given the inducing effect of GPE on matrix mineralization in MSCs during osteogenic differentiation, we next assessed its impact on the expression and activity of markers that promote the initiation and progression of the osteogenic program in AdMSCs. Specifically, we demonstrated that GPE significantly induced the expression of ALP, an early and well-established marker of osteogenic differentiation, as early as 7 days, with a further increase observed at 14 days (Figure 6A). This inductive effect was consistent with the corresponding increase in ALP enzymatic activity (Figure 6B).

**Figure 7:**
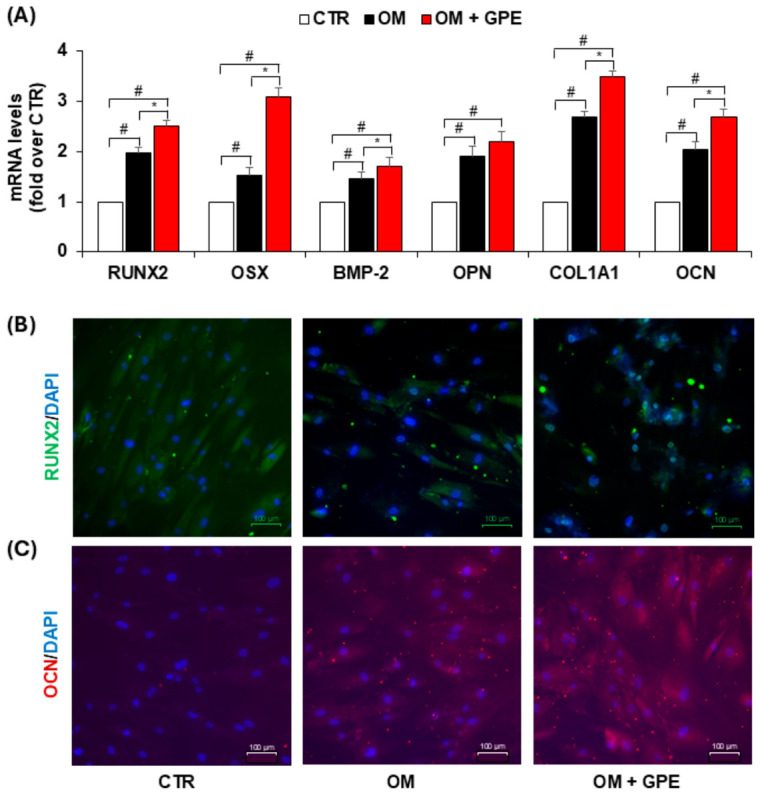
GPE’s effects on osteogenic differentiation markers. AdMSCs were cultured in basal medium (CTR) or osteogenic differentiation medium (OM) with or without GPE (5 µg GAE/mL) for 7 days. The mRNA levels of Runt-related transcription factor 2 (RUNX2), Osterix (OSX), Bone Morphogenetic Protein-2 (BMP-2), Osteopontin (OPN), Collagen Type I Alpha 1 Chain (COL1A1), and Osteocalcin (OCN) were analyzed using quantitative real-time polymerase chain reaction (qRT-PCR) (**A**). RUNX2 nuclear translocation was assessed by immunofluorescent staining by using Alexa Fluor 488-conjugated goat anti-mouse antibody (green fluorescence) and DAPI blue nuclear staining (**B**). Osteocalcin protein expression was assessed by immunofluorescent staining by using Cy3-conjugated goat anti-rabbit (red fluorescence) and DAPI blue nuclear staining (**C**). Scale bar: 100 μm. Each experiment was performed in triplicate. All data are presented as mean  ±  standard deviation (SD). # *p* < 0.01 versus undifferentiated control cells (CTR); * *p* < 0.05 versus osteogenic differentiated cells (OM).

Moreover, the osteoinductive effect of GPE was further supported by a significant increase in the mRNA expression of RUNX2, a key transcription factor regulating osteogenesis (Figure 7A). In addition, immunocytochemical analysis revealed that GPE promoted RUNX2 nuclear translocation. While RUNX2 was predominantly localized in the cytoplasm of undifferentiated control cells, osteogenic induction triggered its nuclear localization, which was further enhanced by GPE treatment (Figure 7B).

The increased nuclear translocation of RUNX2 was associated with the upregulation of several genes involved in the progression of the osteogenic program, including the transcription factor osterix (OSX), BMP-2, osteopontin (OPN), Collagen Type I Alpha 1 Chain (COL1A1), and osteocalcin (OCN). (Figure 7A). Consistently, GPE-induced upregulation of OCN expression was also confirmed by immunocytochemical analysis (Figure 7C).

### 3.5. GPE Activates NRF2 Antioxidant Signaling in AdMSCs

**Figure 8:**
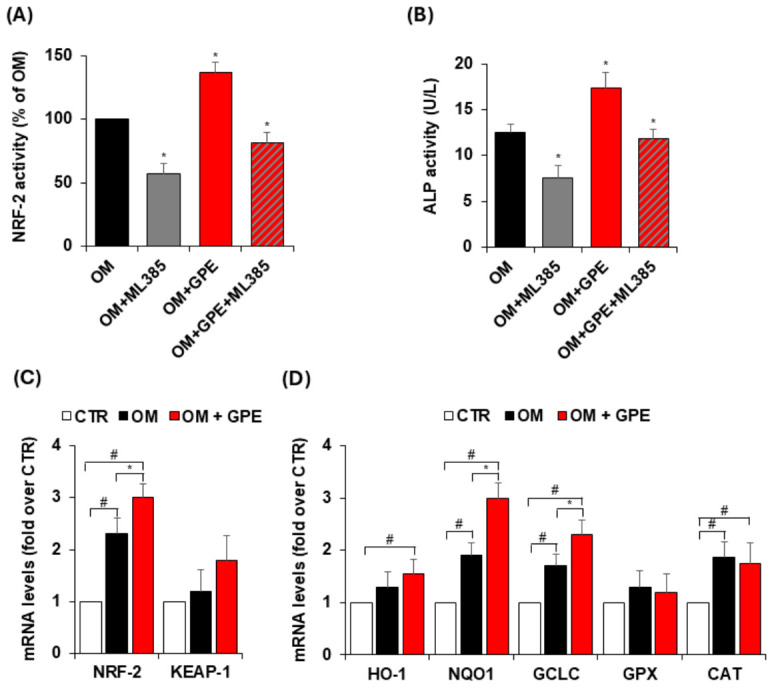
GPE induces NRF2 expression and activity during AdMSC osteogenic differentiation. AdMSCs were cultured in osteogenic differentiation medium (OM) with or without GPE (5 µg GAE/mL) in the presence and absence of the NRF2 inhibitor (ML385, 5 µmol/L). After 3 days, NRF2 translocation was assessed using the TransAM NRF2 DNA-binding ELISA (**A**) and ALP activity (**B**). After 7 days, mRNA levels of NRF2, Kelch-like ECH-associated protein 1 (KEAP1) (**C**), heme oxygenase-1 (HO-1), NAD(P)H quinone dehydrogenase 1 (NQO1), glutamate-cysteine ligase catalytic subunit (GCLC), glutathione peroxidase (GPX) and catalase (CAT) (**D**) were assessed by qRT-PCR. Each experiment was performed in triplicate. All data are presented as mean  ±  standard deviation (SD). # *p* < 0.01 versus undifferentiated control cells (CTR); * *p* < 0.05 versus osteogenic differentiated cells (OM).

Since redox homeostasis critically influences MSC fate and osteogenic differentiation, we next examined whether GPE modulates antioxidant pathways by affecting the expression and activity of NRF2. As shown in Figure 8, osteogenic induction of AdMSCs resulted in NRF2 activation, as evidenced by increased activity of the nuclear NRF2 (Figure 8A).

In our study, NRF2 activity was assessed using the Active Motif TransAM NRF2 assay, which specifically measures the binding of NRF2 to immobilized ARE consensus sequences. Using this approach, we observed a significant increase in nuclear NRF2 DNA-binding activity in AdMSC cultured in osteogenic medium, with a further enhancement following GPE treatment (Figure 8A), indicating increased functional engagement of NRF2 with its target DNA sequences.

To further investigate the role of NRF2 in MSC osteogenic differentiation, AdMSCs were treated with ML385, a well-known NRF2 inhibitor. Cells were exposed to ML385 at a concentration of 5 μg/mL, which did not affect cell viability, as evaluated by MTT assay (Figure S2). As shown in Figure 8A, ML385 effectively inhibited the activation of NRF2 and attenuated the GPE-induced increase in NRF2 activity. In detail, NRF2 activity was reduced by 43% following treatment with the specific NRF2 inhibitor ML385. Notably, GPE significantly increased NRF2 activity by 37% compared with osteogenic medium alone; this effect was completely abrogated in the presence of ML385 (Figure 8A).

The functional relevance of NRF2 activation in GPE-mediated osteogenesis was further confirmed by the suppression of ALP activity upon pharmacological inhibition of NRF2 signaling (Figure 8B). Indeed, ML385 significantly reduced ALP activity under osteogenic conditions, indicating that NRF2 contributes to basal osteogenic differentiation in this model (Figure 8B). Consistently, ML385 blunted the pro-osteogenic effect of GPE, reducing ALP activity to levels comparable to those of osteogenic medium alone (Figure 8B). Overall, these results indicate that NRF2 contributes to osteogenesis and participates in mediating the effects of GPE. Moreover, we observed an increase in NRF2 mRNA levels in AdMSCs under osteogenic conditions, which was further enhanced by GPE treatment, while KEAP1 mRNA expression remained unchanged (Figure 8C). This apparent dissociation is consistent with the current understanding that NRF2 is regulated not only through the canonical KEAP1-dependent pathway but also via transcriptional and post-transcriptional mechanisms.

To evaluate the full transcriptional activation of the NRF2 pathway, we analyzed the expression of several NRF2-regulated antioxidant genes, including HO-1, GPX, CAT, as well as GCLC and NQO1. Our results reported that both osteogenic medium and GPE treatment induced a significant increase or a consistent upward trend in the expression of all analyzed genes compared to control condition (Figure 8C,D). Specifically, osteogenic medium significantly upregulated NQO1, GCLC, and CAT mRNA levels, while HO-1 and GPX showed an increasing trend (Figure 8D). Notably, GPE treatment further enhanced the expression of NQO1, and GCLC compared to osteogenic medium alone, and significantly increased HO-1 and CAT expression compared to control conditions (Figure 8D). Collectively, these findings support the activation of NRF2 signaling in AdMSC in our experimental system.

**Figure 9:**
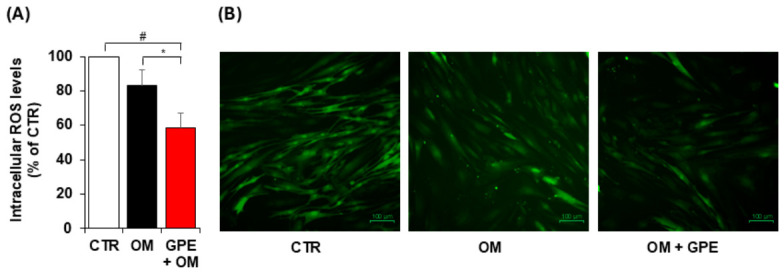
GPE reduces intracellular ROS levels during AdMSC osteogenic differentiation. AdMSCs were cultured in basal medium (CTR) or osteogenic differentiation medium (OM) with or without GPE (5 µg GAE/mL), and after 3 days, the intracellular ROS levels were assessed by using carboxy-H2DCFDA staining by fluorescence plate reader (**A**) and microscopic analysis (**B**), Each experiment was performed in triplicate. All data are presented as mean  ±  standard deviation (SD). # *p* < 0.01 versus undifferentiated control cells (CTR); * *p* < 0.05 versus osteogenic differentiated cells (OM).

Consistent with the observed NRF2 activation, we found that GPE effectively attenuated oxidative stress in AdMSCs undergoing osteogenic differentiation, significantly reducing intracellular ROS levels, as assessed by carboxy-H_2_DCFDA staining (Figure 9).

Having established that GPE activates NRF2 antioxidant signaling in AdMSCs, we next examined whether this mechanism was context-dependent or conserved across MSC sources. Accordingly, we evaluated the effects of GPE on NRF2 activation in canonical BMSCs. Consistent with the findings observed in AdMSCs, GPE was also shown to modulate NRF2 signaling during BMSC osteogenesis, significantly enhancing NRF2 activity and upregulating the expression of NRF2-regulated antioxidant genes, including NQO1, GCLC, and CAT (Figure S3). Overall, these findings suggest that GPE exerts a conserved mechanism of action in the osteogenic differentiation of MSCs, irrespective of their origin.

## 4. Discussion

Osteoporosis is a highly prevalent age-related chronic disease, primarily affecting postmenopausal women, and is characterized by an imbalance between bone resorption and formation, leading to reduced bone density and fragility. Although effective pharmacological treatments are available, their clinical use is often limited by adverse effects and restricted treatment duration. Consequently, increasing attention has been directed toward natural and safe compounds as complementary or alternative strategies for the prevention of bone loss. Among these, polyphenols have attracted considerable interest due to their influence on bone metabolism and remodeling [7,19]. Consistently, in vitro and in vivo studies support a positive association between dietary polyphenol intake and bone health [7,20], and suggest nutritional strategies based on polyphenol-rich foods as adjuvants to conventional therapies to reduce osteoporosis progression and fracture risk [7,19,21].

Grape pomace, a major by-product of winemaking, has been extensively investigated for its antioxidant, anti-inflammatory, cardioprotective, and anti-ageing activities. Notably, grape pomace polyphenols have been reported to exert protective and therapeutic effects in osteoporosis, bone necrosis, and inflammatory autoimmune arthritis, as well as to improve bone density and mechanical strength [7,22]. However, the phytochemical composition of grape pomace varies depending on grape cultivar, vinification procedures, and extraction methods, which may significantly influence its biological activity [23].

In the present study, we aimed to investigate whether a sustainable, polyphenol-rich extract obtained from red grape pomace could modulate MSC fate and restore the osteogenic/adipogenic balance implicated in osteoporosis and age-related bone loss. Within the framework of circular economy and green chemistry principles, we obtained a polyphenol-rich extract from an aqueous infusion of Negroamaro red grape pomace. The resulting GPE was characterized by high levels of anthocyanins, phenolic acids, flavanols, stilbenes, and flavonols, and exhibited marked antioxidant activity in a cell-free system. Functional analyses revealed a clear osteoinductive effect of GPE, which significantly promoted BMSC differentiation toward the osteoblastic lineage while simultaneously suppressing adipogenic differentiation, without compromising cell viability. Notably, this dual activity was consistently observed not only in canonical BMSC models but also in AdMSCs isolated from adipose tissue of elderly subjects (>65 years) with cardiovascular disease, a clinical context often associated with impaired MSC function [24,25,26,27]. The anti-adipogenic effect was supported by the reduced expression of key transcriptional regulators of the adipogenic program. Specifically, GPE significantly reduced the expression of the master transcription factor PPARγ, a pivotal driver of adipocyte commitment, together with its downstream target genes. GPE markedly downregulated CD36, a transmembrane transporter that facilitates fatty acid uptake, and FABP4, an intracellular lipid chaperone involved in fatty acid trafficking and storage. Importantly, these effects were paralleled by a relevant enhancement of osteogenic activity and marker expression. GPE increased ALP expression and activity in the early stages of osteogenic differentiation, maintaining elevated levels over time, indicating a sustained pro-osteogenic effect. The osteoinductive activity of GPE was further substantiated by the upregulation of RUNX2, the master transcription factor governing osteoblast commitment and MSC osteogenic differentiation. The pivotal role of RUNX2 in skeletal development is well established, its deficiency results in the absence of a mineralized skeleton, whereas its overexpression initiates and amplifies the osteogenic transcriptional program [28]. In addition to increasing total RUNX2 expression, GPE promoted its nuclear translocation in AdMSCs undergoing osteogenic differentiation, indicating functional activation of the transcription factor. The enhanced nuclear localization of RUNX2 by GPE was associated with the coordinated upregulation of osteogenic markers at different stages of differentiation. Early markers such as osterix and BMP-2, as well as late markers including osteopontin, COL1A1, and osteocalcin, were significantly increased. Mechanistically, RUNX2 promotes osteoblast differentiation by activating genes encoding key matrix proteins as well as the transcription factor osterix crucial for terminal maturation and mineralization [29,30].

Osteogenic differentiation and bone formation depend on the tightly regulated production of reactive oxygen species (ROS). Although physiological ROS levels are required for cellular signaling, excessive ROS can impair osteogenesis and induce cellular damage. Cells possess sophisticated antioxidant systems, including the transcription factor NRF2, that tightly regulate ROS production and detoxification, thereby preserving redox homeostasis. In bone biology, NRF2 exerts a complex role dependent on specific microenvironments. Moderate activation of NRF2 supports osteoblast differentiation and mineralization—partly through the regulation of osteogenic transcription factors such as RUNX2—while simultaneously inhibiting osteoclastogenesis and bone resorption. Conversely, both NRF2 deficiency and excessive activation have been shown to impair bone homeostasis, highlighting the importance of tightly balanced NRF2 signaling [3,4]. Owing to this dual role, NRF2 has emerged as a promising therapeutic target for bone-related disorders, including osteoporosis.

Given the well-established antioxidant properties of grape pomace polyphenols [12,18,31], this study aimed to investigate the potential role of GPE in modulating oxidative stress and activating the NRF2 signaling pathway during the osteogenic differentiation of human MSCs. We demonstrate that GPE activates NRF2-dependent antioxidant signaling by promoting NRF2 nuclear translocation and transcriptional activity in AdMSCs. We found that GPE affects the expression of several NRF2-regulated antioxidant genes, including HO-1, GPX, CAT, GCLC, and NQO1. NRF2 activation was associated with increased NRF2 mRNA expression, with no changes in KEAP1 mRNA levels. This finding is consistent with current knowledge that NRF2 can be regulated not only through the canonical KEAP1-dependent pathway, but also through transcriptional and post-transcriptional mechanisms. Specifically, under conditions of moderate oxidative stress, such as those occurring during osteogenic differentiation, NRF2 can be upregulated through redox-sensitive transcriptional pathways and positive feedback mechanisms. Moreover, pharmacological inhibition of NRF2 signaling significantly attenuated GPE-induced ALP activity during osteogenic differentiation, indicating that NRF2 activation is a critical upstream mediator of the osteogenic response to GPE.

The osteoinductive potential of grape pomace polyphenols has previously been reported by Torre et al. [32], who showed that extracts obtained from different grape varieties promoted BMSC differentiation toward the osteoblastic lineage through upregulation of key osteogenic markers. Consistent with these observations, our findings confirm the pro-osteogenic activity of grape pomace polyphenols in BMSCs and further extend the osteoinductive effect of GPE to adipose tissue–derived MSCs. Our results also align with a substantial body of evidence indicating that the major polyphenol classes enriched in GPE exert coordinated dual effects on MSC differentiation. Anthocyanins, prominent constituents of GPE, have been shown to suppress adipogenic commitment by downregulating the master transcription factors PPARγ and C/EBPα, reducing FABP4 expression, and limiting intracellular lipid accumulation [33,34]. In parallel, specific anthocyanins such as delphinidin enhance osteogenic differentiation by increasing RUNX2, ALP, and mineral deposition, partly via BMP and AMPK signaling pathways [35,36]. Flavanols such as catechins similarly inhibit adipogenesis through AMPK activation and repression of PPARγ transcriptional activity [37,38]. Beyond their anti-adipogenic action, catechins promote osteoblast differentiation in MSCs by increasing ALP activity, calcium deposition, and the expression of osteogenic genes including RUNX2 and osteocalcin, effects associated with AMPK activation and protection against oxidative stress [39]. Among flavonols, quercetin has been widely reported to suppress adipogenic differentiation via inhibition of PPARγ, C/EBPα, and FABP4 [40,41,42]. Concomitantly, quercetin enhances osteogenic commitment and maturation by activating ERK/MAPK and BMP pathways, resulting in increased expression of RUNX2, Osterix, ALP, and osteocalcin, as well as enhanced mineralization [42]. Stilbenes such as resveratrol and its glucoside precursor polydatin further reinforce this dual regulatory effect. Resveratrol inhibits adipogenesis through activation of the SIRT1–AMPK axis and repression of PPARγ signaling [43,44], while promoting osteogenic differentiation of MSCs by upregulating RUNX2, osteocalcin, and ALP, and enhancing matrix mineralization [45,46,47,48]. Phenolic acids, including gallic acid and p-coumaric acid, also exhibit anti-adipogenic activity associated with reduced lipid accumulation and downregulation of adipogenic markers [49,50,51]. In parallel, these compounds have been reported to support osteogenic differentiation by enhancing RUNX2, COL1A1, and ALP expression and promoting matrix mineralization, effects largely attributed to their antioxidant properties and modulation of redox-sensitive signaling pathways.

Overall, this study demonstrates the osteoinductive potential of a polyphenol-rich extract derived from red grape pomace, reporting robust bioactivity not only in BMSCs but also in AdMSCs from elderly individuals at high cardiovascular risk. This aspect is particularly relevant from a clinical perspective, as chronic inflammation and oxidative stress, key drivers of atherosclerotic cardiovascular disease, also contribute significantly to the pathogenesis of osteoporosis. The ability of GPE to restore the osteogenic/adipogenic balance of MSCs in this context suggests a potential dual benefit in conditions where skeletal fragility and cardiometabolic dysfunction often coexist.

However, several limitations must be acknowledged. The study was conducted exclusively in vitro and therefore does not fully capture the complexity of the bone microenvironment, systemic metabolic interactions, or bioavailability constraints that may influence the biological activity of polyphenols in vivo. Furthermore, the precise contribution of individual polyphenolic compounds within the extract, as well as their potential synergistic interactions, remains to be elucidated. Future studies in appropriate animal models and in clinical settings will be essential to determine the pharmacokinetic, efficacy, and safety profile of GPE and to establish its translational potential as a nutritional or adjunctive strategy for the prevention of age-related bone loss.

## 5. Conclusions

In summary, this study demonstrates that polyphenol-rich extracts obtained from the aqueous infusion of red grape pomace reduce oxidative stress while promoting osteogenesis, with particularly pronounced effects in adipose-derived mesenchymal stem cells from elderly subjects at high cardiovascular risk.

These findings support the potential of grape pomace-derived polyphenols as the basis for a functional beverage with translational relevance, representing a promising nutritional strategy to counteract age-related bone fragility, even in the presence of comorbidities that are known to impair mesenchymal stem cell function.

## Figures and Tables

**Table 1:** Oligonucleotides used for quantitative real-time PCR analysis.

Gene Symbol	Full Name	Forward Primer (5′-3′)	Reverse Primer (5′-3′)
PPARy	Peroxisome proliferator-activated receptor γ	tgcaggtgatcaagaagacg	agtgcaactggaagaaggga
CD36	CD36 molecule	agatgcagcctcatttccac	gccttggatggaagaacaaa
FABP4	Fatty acid-binding protein 4	gtggaagtgacgcctttcat	tactgggccaggaatttgac
ALP	Alkaline phosphatase	ttgacctcctcggaagacactctg	cgcctggtagttgttgtgagcatag
RUNX2	Runt related transcription factor 2	gacaaccgcaccatggtgg	tctggtacctctccgaggg
OCN	Osteocalcin	gctacctgtatcaatggct	cgatgtggtcagccaactc
OPN	Osteopontin	cccacagacccttccaagta	ggggacaactggagtgaaaa
OSX	Osterix	aattgccaggagctagagcg	ctggtgtttgctcaggtggt
COL1A1	Collagen type I alpha 1 chain	agggaatgcctggtgaacg	gagagccatcagcacctttg
BMP-2	Bone morphogenetic protein-2	agacctgtatcgcaggcact	cctccgtggggatagaactt
NRF2	Nuclear factor erythroid 2-related factor 2	gcgacggaaagagtatgagc	gttggcagatccactggttt
KEAP1	Kelch-like ECH-associated protein 1	ccttcagctacaccctggag	catgaccttggggtggatac
HO-1	Heme-oxygenase-1	cttcttcaccttccccaaca	cctgcaactcctcaaagagc
CAT	Catalase	tggaaagaagactcccatcg	ccagaagtcccagaccatgt
GPX	Glutathione peroxidase	ttgacatcgagcctgacatc	ctgacacccggcactttatt
GCLC	Glutamate-cysteine ligase catalytic subunit	accatcatcaatgggaagga	gcgataaactccctcatcca
NQO1	NAD(P)H quinone dehydrogenase 1	gcactgatcgtactggctca	cgcagggtccttcagtttac
GAPDH	Glyceraldehyde-3-phosphate dehydrogenase	atcactgccacccagaagac	ttctagacggcaggtcaggt

**Table 2:** Characterization of polyphenolic classes of GPE.

Polyphenolic Classes	(μg/g)
**Anthocyanins**	452.07 ± 9.63
**Phenolic Acids**	436.33 ± 1.88
**Flavanols**	240.89 ± 2.96
**Stilbenes**	121.03 ± 2.75
**Flavonols**	107.33 ± 4.86

Data are expressed as mean value ± SD and are representative of 3 independent experiments.

## Data Availability

The data supporting this article have all been included in the text.

## Supplementary Materials

The following supporting information can be downloaded at https://www.mdpi.com/article/10.3390/biology15090719/s1: Figure S1: Effects of GPE on human mesenchymal stem viability. Figure S2: Effects of GPE on human mesenchymal stem viability. Figure S3: GPE induces NRF2 activity during BMSC osteogenic differentiation.

## Abbreviations

MSC	Mesenchymal stem cell
BMSCs	Bone-marrow mesenchymal stem cells
AdMSCs	Adipose tissue-derived mesenchymal stem cells
GPE	Grape pomace extract
OM	Osteogenic medium
AM	Adipogenic medium
MTT	Thiazolyl blue tetrazolium bromide
CM-H2DCFDA	Carboxy-2,7-dichlorofluorescein diacetate
ARS	Alizarin Red S
ORO	Oil Red O
PPARy	Peroxisome proliferator-activated receptor γ
CD36	CD36 molecule
FABP4	Fatty acid-binding protein 4
ALP	Alkaline phosphatase
RUNX2	Runt related transcription factor 2
OCN	Osteocalcin
OPN	Osteopontin
OSX	Osterix
COL1A1	Collagen type I alpha 1 chain
BMP-2	Bone morphogenetic protein-2
NRF2	Nuclear factor erythroid 2-related factor 2
KEAP1	Kelch-like ECH-associated protein 1
HO-1	Heme-oxygenase-1
CAT	Catalase
GPX	Glutathione peroxidase
GAPDH	Glyceraldehyde-3-phosphate dehydrogenase
