# Supplementary material for: Grape Pomace Polyphenolic Extract Promotes Osteogenic Differentiation in Human Mesenchymal Stem Cells Through Activation of RUNX2 and NRF2 Transcription Factors: A Potential Natural Strategy for Osteoporosis Prevention
Source: Biology (Basel). 2026 May 1;15(9):719. doi: 10.3390/biology15090719 (PMC13163114; doi:10.3390/biology15090719)
Supplement: Supplementary file 1 [file biology-15-00719-s001.zip › biology-4237351-supplementary.pdf]

biology

MDPI

Supplementary Materials

Grape Pomace Polyphenolic Extract Promotes Osteogenic dDiffer-Entiation in Human Mesenchymal Stem Cells Through Activation of RUNX2 and NRF2 Transcription Factors: A Potential Natural Strategy for Osteoporosis Prevention

Nadia Calabriso [1,*], Marika Massaro [1], Stefano Quarta [1,2], Luisa Siculella [2], Giuseppe Santarpino [3], Tiziano Verri [4], Carmela Gerardi [5], Giovanna Giovinazzo [5] and Maria Annunziata Carluccio [1]

1 Institute of Clinical Physiology (IFC), National Research Council (CNR), 73100 Lecce, Italy
2 Department of Experimental Medicine (DiMeS), University of Salento, 73100, Lecce, Italy
3 Department of Clinical and Experimental Medicine, Magna Graecia University of Catanzaro, Italy
4 Department of Biological and Environmental Sciences and Technologies (DISTEBA), University of Salento, 73100 Lecce, Italy
5 Institute of Sciences of Food Production (ISPA), National Research Council (CNR), 73100 Lecce, Italy

* Correspondence: nadia.calabriso@cnr.it

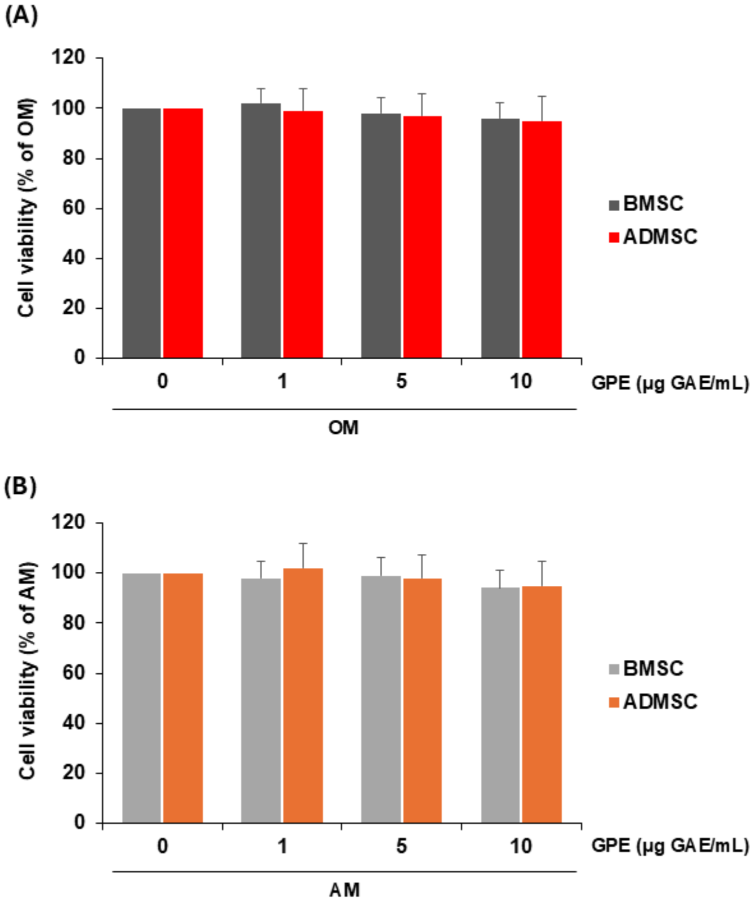

Figure S1. Effects of GPE on human mesenchymal stem viability. BMSC and AdMSC were exposed at increasing concentrations of GPE (1, 5 and 10 μg GAE/mL) in presence of osteogenic medium (A) or adipogenic medium (B) for 24 h and cell viability was evaluated by MTT assay. Data are shown as mean ± SD (n = 5).

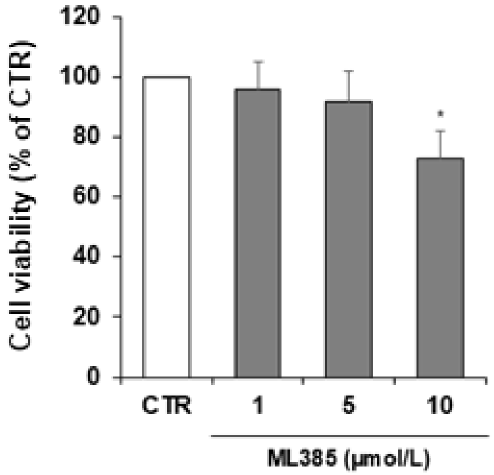

Figure S2. Effects of GPE on human mesenchymal stem viability. AdMSC were exposed at increasing concentrations of ML385 (1, 5 and 10 µmol/L) for 24 h and cell viability was evaluated by MTT assay. Data are shown as mean ± SD (n = 5); *p < 0.05 versus untreated control cells (CTR).

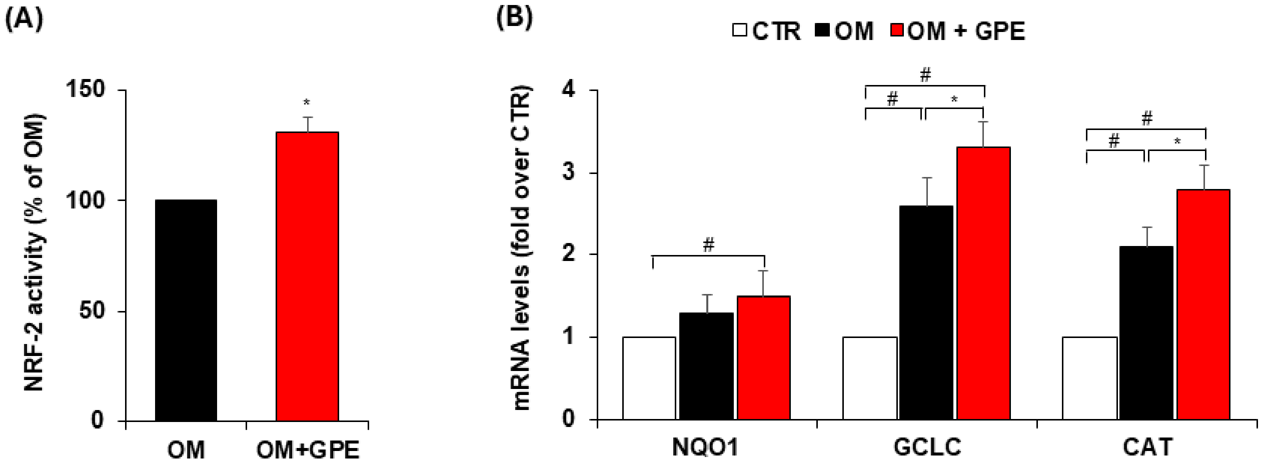

Figure S3. GPE induces NRF2 activity during BMSC osteogenic differentiation. BMSCs were cultured in osteogenic differentiation medium (OM) with or without GPE (5 µg GAE/mL). After 3 days, NRF2 translocation was assessed using the TransAM NRF2 DNA-binding ELISA (A). After 7 days, mRNA levels of NAD(P)H quinone dehydrogenase 1 (NQO1), glutamate-cysteine ligase catalytic subunit (GCLC) and catalase (CAT) were assessed by qRT-PCR (B). Each experiment was performed in triplicate. All data are presented as mean ± standard deviation (SD). #p<0.01 versus undifferentiated control cells (CTR); *p<0.05 versus osteogenic differentiated cells (OM).
